# Early Detection of Cardiotoxicity From Systemic and Radiation Therapy in Patients With Breast Cancer: Protocol for a Multi-Institutional Prospective Study

**DOI:** 10.2196/31887

**Published:** 2022-04-21

**Authors:** Giulia Borgonovo, Elen Vettus, Alessandra Greco, Laura Anna Leo, Francesco Fulvio Faletra, Catherine Klersy, Moreno Curti, Mariacarla Valli

**Affiliations:** 1 Clinic of Radiation Oncology Oncology Institute of Southern Switzerland Bellinzona Switzerland; 2 Department of Oncology East Tallinn Central Hospital Tallinn Estonia; 3 Division of Cardiology Fondazione Istituto di Ricovero e Cura a Carattere Scientifico Policlinico San Matteo Pavia Italy; 4 Department of Cardiology Fondazione Cardiocentro Ticino Lugano Switzerland; 5 Fondazione Istituto di Ricovero e Cura a Carattere Scientifico Policlinico San Matteo Pavia Italy

**Keywords:** breast cancer, cardiotoxicity, cardiac diagnostic imaging, radiotherapy, chemotherapy

## Abstract

**Background:**

The incidence of breast cancer is rising worldwide. Recent advances in systemic and local treatments have significantly improved survival rates of patients having early breast cancer. In the last decade, great attention has been paid to the prevention and early detection of cardiotoxicity induced by breast cancer treatments. Systemic therapy-related cardiac toxicities have been extensively studied. Radiotherapy, an essential component of breast cancer treatment, can also increase the risk of heart diseases. Consequently, it is important to balance the expected benefits of cancer treatment with cardiovascular risk and to identify strategies to prevent cardiotoxicity and improve long-term outcomes and quality of life for these patients.

**Objective:**

This CardioTox Breast study aims to investigate the use of cardiac imaging, based on cardiac magnetic resonance and echocardiography, and to identify associated circulating biomarkers to assess early tissue changes in chemo-induced and radiation-induced cardiotoxicity in the time window of 12 months after the end of radiotherapy in patients with breast cancer.

**Methods:**

The CardioTox Breast trial is a multicenter observational prospective longitudinal study. We aim to enroll 150 women with stage I-III unilateral breast cancer, treated with breast conserving surgery, who planned to receive radiotherapy with or without systemic therapy. Baseline and follow-up data include cardiac measurements based on cardiac magnetic resonance imaging, echocardiography, and circulating biomarkers of cardiac toxicity.

**Results:**

This study details the protocol of the CardioTox Breast trial. Recruitment started in September 2020. The results of this study will not be published until data are mature for the final analysis of the primary study end point.

**Conclusions:**

The CardioTox Breast study is designed to investigate the effects of systemic and radiation therapy on myocardial function and structure, thus providing additional evidence on whether cardiac magnetic resonance is the optimal screening imaging for cardiotoxicity.

**Trial Registration:**

ClinicalTrials.gov NCT04790266; https://clinicaltrials.gov/ct2/show/NCT04790266

**International Registered Report Identifier (IRRID):**

DERR1-10.2196/31887

## Introduction

Incidence of breast cancer is rising worldwide. Due to developments in systemic and local therapies, median survival of early breast cancer patients has increased [1], resulting in decreased risk of local recurrence and breast cancer death, as well as longer life expectancy [2-4]. Consequently, the reduction in treatments’ side effects and the improvement of patients’ quality of life are becoming increasingly important in treatment prescription and planning. The most frequent toxicities described in literature are neurological, hematological, gastrointestinal, cutaneous, and cardiological ones. In the last decade, great attention has been paid to prevention and early detection of cardiotoxicity because heart diseases are the main nononcological cause of death in these patients [5].

Our understanding of the pathophysiology and natural history of iatrogenic cardiotoxicity remains limited, and the diagnosis is carried out frequently only when cardiovascular disease presents clinically [6]. Cardiovascular complications from cancer therapies are widely heterogeneous (eg, myocardial dysfunction, heart failure [HF], coronary artery disease, valvular disease, arrhythmias, and arterial hypertension). Myocardial dysfunction and HF are the most frequent complications after breast cancer treatments. It is believed that cardiotoxicity is a continuous phenomenon beginning with myocardial injury and changes in myocardial strain; subsequently, a progressive left ventricular ejection fraction (LVEF) decline may gradually lead to symptomatic HF [7]. If HF treatment is delayed, it is possible that the cardiac function may never restore to what it was at baseline [8]. It is therefore fundamental to distinguish asymptomatic patients who are at risk for cardiotoxicity and, if possible, give them protective treatment for avoiding side effects.

Therefore, cardio-oncology is a rapidly developing subspecialty within cardiology, which aims to optimize the diagnosis and management of cancer treatment cardiac complications [9].

The American Society of Echocardiography and the European Association of Cardiovascular Imaging define cardiotoxicity as a decline of LVEF ≥10% with a final LVEF <53%.

Anthracyclines-based systemic therapy is considered as the prototype of type I cardiotoxic agents [10]. It is believed that agents belonging to this group cause irreversible continuous progressive decline in LVEF, which is dose dependent and can lead to dilated cardiomyopathy [11]. Anthracycline-induced cardiotoxicity may present during or immediately after the infusion (acute), within the first year of treatment (early) and years after treatment (late) [12,13].

Trastuzumab, an anti-human epidermal growth factor receptor 2 (HER2) agent is considered a standard treatment in breast cancer overexpressing HER2. As a type II cardiotoxic agent, it induces cardiotoxicity that is usually reversible with its interruption or with HF treatment. Toxicity from type II agents is not related to cumulative dose and usually develops during treatment [11].

The incidence of anthracycline-related cardiotoxicity is 3%-48% while it varies from 1.7% to 20.1% with trastuzumab.

Due to their potential cardiotoxicity, anthracycline and trastuzumab are usually not administered concurrently [11].

Radiotherapy (RT) can also increase the risk of several heart diseases [14]. The relative risk varies from 3.5 (for left-sided) to 1.2 (for right-sided) breast cancer [15]. Radiation-induced cardiotoxicity usually develops even more than 10 years after RT with an interstitial myocardial fibrosis [8,15]. RT may induce ischemic heart disease through the development of severe atherosclerotic and nonatherosclerotic disease, complicated by plaque rupture and thrombosis, and potentially with coronary spasm [11,16]. Cardiac damage has a strict correlation with the mean heart dose, with a 7.4% increase in relative risk of major cardiac event for each additional 1 Gy of mean heart dose [15]. The mean heart dose has to be as low as possible, almost 0 Gy, albeit at the critical portions of the heart, as left ventricle or left anterior descending artery can receive more than 40 Gy in a very limited volume [17]. Technological developments in RT techniques such as intensity-modulated RT or volumetric modulated arc therapy and deep inspiration breath hold have allowed a reduction in cardiac doses, particularly for patients with left-sided breast cancer, lowering the risk of cardiotoxicity.

A synergistic effect on cardiac risk between left breast RT and cardiotoxic chemotherapy has been described even if its actual incidence is difficult to quantify and evaluate.

Cardiotoxicity can be detected by different diagnostic methods. Circulating biomarkers could detect and predict cardiotoxicity. The 2014 American Society of Echocardiography guidelines the recommend measurement of troponin at baseline, before systemic therapy and 24 hours after, to aid in the detection of subclinical cardiotoxicity.

Troponin and brain natriuretic peptide (BNP) have been investigated in many trials, but unfortunately with different results. Several studies have shown that the elevation of Troponin I may predict the development of future LVEF depression, and N-terminal pro b-type natriuretic peptide can predict the risk for radiation-induced cardiotoxicity [7,8]. Of note, 1 study has showed that a reduction in longitudinal strain and an increase in high-sensitivity troponin, after the end of anthracycline therapy, predicted future left ventricular dysfunction [7].

Several epidemiologic studies have shown a significant association between elevated plasma concentrations of high-sensitivity C-reactive protein (hs-CRP) and the prevalence of underlying atherosclerotic vascular disease, as well as the risk of recurrent cardiovascular events among patients with established disease and apparently healthy individuals [18-20].

In 2012, Onitilo and his colleagues [21] published a pilot study about hs-CRP as a biomarker for trastuzumab-induced cardiotoxicity. They showed that abnormal hs-CRP (≥3 mg/L) predicted decreased LVEF with a sensitivity of 92.9%.

Cardiac biomarkers could also be potential candidates to monitor cardiac damage after RT. Echocardiography (ECHO) is currently the standard method for detecting cardiotoxicity, usually by monitoring serial LVEF. A 3-dimensional modality is preferred to a 2-dimensional one because of better reproducibility [22]. LVEF is not directly correlated to early toxicity and usually decreases months after myocardial cell injury has happened. A recent advanced echocardiographic technique, automated 2-dimensional speckle tracking echocardiography (cardiac strain), has been used for detecting and quantifying subclinical changes in left ventricular strain and function. The use of global longitudinal strain (GLS) by speckle tracking echocardiography is strongly recommended because of its feasibility and biological reproducibility [22]. GLS is changing earlier than LVEF, corresponding to myocardial deformation; consequently, this technique could diagnose cardiotoxicity more quickly [8,23].

Current guidelines suggest ECHO at baseline, after the end of anthracycline therapy, before the start of trastuzumab treatment, and every 3 months during trastuzumab treatment.

Cardiac magnetic resonance (CMR) is the most accurate methodology for the evaluation of volumes and function of heart chambers. Additionally, it is exceptionally capable of providing myocardial tissue characterization, including the presence and extension of myocardial oedema (reflected in increased T2-weighted magnetic resonance imaging), hyperemia (assessed by an increment in early enhancement), and fibrosis (visualized with late enhancement techniques) [24].

Serial CMR imaging showed reduction in LVEF, 12 to 24 months after therapy, in women treated for breast cancer with anthracycline-based chemotherapy. Few recent studies have suggested that LVEF could start to decline earlier; however, the prognostic implications of these changes are not yet known. Recent preliminary experimental data suggest that the decline in contractile function is preceded by CMR evidence of myocardial oedema with T2 (transverse relaxation time) sequences and T2 mapping [25-27]. Furthermore, T1 (longitudinal relaxation time) mapping is a promising technique to quantify morphologic tissue injuries, such as interstitial or diffuse myocardial fibrosis. If treated with radiation therapy, patients diagnosed with breast cancer have an increased risk of acute asymptomatic pericardial effusion, which can also be detected by CMR. CMR is the most sensitive and reproducible measure of LVEF.

When biomarkers and cardiac imaging methods (eg, GLS on ECHO and oedema on CMR) are integrated, we could detect preclinical cardiotoxicity and prevent left ventricular dysfunction.

Unfortunately, thus far, these studies have had a small sample size, and therefore we cannot draw any practice changing conclusion.

In our opinion, it is important to better define high-risk patients who need intensive cardiovascular screening during and after cardiotoxic treatment. Our purpose is to detect toxicity when it is still subclinical and reversible and to prevent its deterioration with protective drugs administered to “the right patient at the right time.”

## Methods

### Study Design

This CardioTox Breast study (registered with ClinicalTrials; registration number NCT04790266) is a multicenter, observational, prospective, longitudinal study that includes female patients with left-breast and right-breast cancer treated with postoperative RT, with or without chemotherapy or hormonal therapy after primary breast conservative surgery. The patients will be followed for at least 1 year after RT, with cardiac imaging and circulating biomarkers (further detailed below). Three investigating centers are involved in the study: the Oncology Institute of Southern Switzerland (Bellinzona, Switzeralnd), the North Estonia Medical Center (Tallinn, Estonia), and Fondazione IRCCS Policlinico San Matteo (Pavia, Italy). Furthermore, Cardiocentro Ticino is involved in the study as the center of analysis of CMR and echocardiography in Switzerland.

### Study Population

We aim to enroll 150 female patients aged ≥18 years with stage I-III unilateral breast cancer treated with breast conserving surgery and planned to receive radiation therapy with or without systemic therapy.

### Eligibility Criteria

Inclusion criteria are as follows: (1) written informed consent must be obtained before any assessment is performed; (2) female, aged ≥18 years at visit 1; (3) performance status Eastern Cooperative Oncology Group (ECOG) 0-1; (4) stage I-III histology proven breast cancer (Swiss center allows inclusion of ductal carcinoma in situ); (5) adjuvant RT and neoadjuvant anthracycline and/or trastuzumab-based therapy with or without hormonal therapy; and (6) negative pregnancy test (plasma human chorionic gonadotropin [hCG]) for all female participants of childbearing potential (ie, not permanently sterilized—posthysterectomy or tubal ligation status).

Exclusion criteria are as follows: (1) known metastatic spread of any cancer; (2) known active or recurrent hepatic disorder (cirrhosis, hepatitis), aspartate aminotransferase to alanine aminotransferase ratio of 2 x upper limits of normal; (3) renal function decrease (estimated glomerular filtration rate <30 ml/min); (4) known coronary artery disease; (5) angina pectoris; (6) positive or missing pregnancy test (pre- and perimenopausal women) at enrollment visit; (7) patients with baseline LVEF <53% and GLS <15%; and (8) patients with pacemaker.

### Pretreatment Evaluation

The patients will be recruited in participating centers. The investigators ensure that women will meet the inclusion criteria in the study and sign the written informed consent. On inclusion, patient demographics and proper medical history, including current medications and family history of cardiovascular disease will be recorded. A physical examination will be performed, including the measurement of blood pressure and pulse rate.

A baseline assessment will be conducted before the start of adjuvant treatment by checking CMR, ECHO, an electrocardiogram to detect any arrhythmia, and blood sampling to analyze circulating biomarkers.

### CMR Protocol

CMR will be performed with Siemens Skyra 3T or a 1.5T scanner. All patients will undergo a standard protocol, including late gadolinium enhancement for estimated glomerular filtration rate >30 ml/min. For native T1 and postcontrast mapping, basal, midventricular, apical short axis, and 4-4-3 and 2 chamber apical images will be acquired by “ECG-triggered Modified Look-Locker Inversion recovery” sequence. Additionally, T2 mapping (T2-prepared True-FISP [fast imaging with steady-state free precession]) will be acquired on the same plane. The CMR images and maps will be analyzed offline. Late gadolinium enhancement will be quantified on short-axis stacks using a semiautomatic approach.

### Echocardiography

Transthoracic echocardiography will be performed by using a Philips Epiq or General Electric Vivid E95. Images will be digitally stored for offline analysis on custom software. We will collect the parameters to detect myocardial dysfunction and deformation.

### Blood Sampling Procedures and Biochemical Assays

Blood sampling will be carried out to analyze circulating biomarkers of cardiac injury. High-sensitivity cardiac troponin T, high-sensitivity cardiac troponin I, N-terminal pro-BNP, and inflammatory and anti-inflammatory mediators such as hs-CRP will be measured.

### Treatment

#### Chemotherapy

patients will receive adjuvant chemotherapy in accordance with the international guidelines. Patients receiving cardiotoxic chemotherapy will be drawn blood before and, if possible, 24 hours after chemotherapy administration.

Patients who receive anthracycline have an ECHO and electrocardiography after the end of this schedule.

During trastuzumab, blood sample will be taken before every administration (every 3 weeks), and ECHO will be conducted after every 4 cycles (every 3 months).

#### Radiation Therapy

Patients will undergo a simulation chest computed tomography (CT) in free breathing and breath hold modality. Clinical target volume, the planning target volume, and the organs at risk will be drawn on the breath hold modality imaging if this modality improves the distance between breast and heart or left coronary artery (left anterior descending artery). An appropriate physical dosimetric study will be processed using treatment planning system and will be generated to achieve maximum coverage for the planning target volume region by minimizing the dose to the surrounding healthy tissues.

For each contoured volume, we will analyze the minimum, maximum, and mean dose, and dose volume histogram will be evaluated for obtaining the best treatment.

#### Follow-up

**Figure 1:**
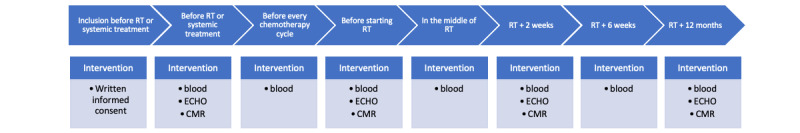
Protocol flow chart description. Blood: blood sample for circulating biomarkers; CMR: cardiac magnetic resonance; ECHO: echocardiography; RT: radiotherapy.

During follow-up, imaging (CMR and ECHO) and biomarkers will be checked 2 weeks after the end of RT and 1 year later. If a patient has symptomatic heart failure during the treatment, or if LVEF declines greater than 10% with a final LVEF <53% on ECHO, the patient will be referred to the cardiologist for a specific treatment. Adverse events will be classified according to the Common Terminology Criteria for Adverse Events. Figure 1 describes the protocol flow chart of the CardioTox Breast study.

### Data Collection

Data will be collected in a dedicated database. An accurate patient history, the type of cancer, and information on the main risk factors for a cardiac event are collected at the inclusion time. Cardiac imaging data (ECHO and CMR) and circulating biomarkers measurements are registered in the database.

### Ethical Considerations

This study will be conducted in accordance with the Declaration of Helsinki (amended at the 64th World Medical Association General Assembly, Fortaleza, Brazil, October 2013) and in accordance with the principles of “Good Clinical Practice” and the Medical Research Involving Human Subjects Act. This study was approved by the ethics committee of the Oncology Institute of Southern Switzerland (ID 2019-01395) and by the local ethical committees of the other 2 investigating centers.

### Study End Points

#### Primary End Point

The primary end point is the incidence of cardiotoxicity, as defined by consensus guidelines (decline of LVEF ≥10% points with a final LVEF of <53%), measured on CMR and ECHO over the time window of 12 months from the end of radiation therapy.

#### Primary Objective

The primary objective is to assess the role of myocardial oedema on CMR (T2 mapping) after radiation and cardiotoxic systemic therapy in predicting the incidence of cardiotoxicity, as defined by consensus guidelines (decline of LVEF ≥10% points with a final LVEF <53%), measured on CMR and ECHO over the time window of 12 months from the end of radiation therapy.

#### Secondary Objectives

The secondary objectives are the following: (1) detect GLS decrease >15% from baseline, measured on ECHO over the time window of 12 months; (2) see if the changes in biomarkers will correlate with LVEF measurements, assessed by ECHO and CMR; (3) see if the changes in biomarkers will correlate with GLS measurements, assessed by ECHO; (4) compare the time to the biomarkers’ positivity to the time to decrease in GLS >15% or decline of LVEF ≥10% with a final LVEF of <53% measured on ECHO; (5) find out if patients with increased baseline biomarkers will develop cardiotoxicity, and identify predictors of cardiotoxicity by multivariable analysis; (6) detect major cardiovascular events (defined as acute myocardial infarction, hospitalization due to heart failure, atrial flutter or fibrillation, and ventricular tachycardia) or death due cardiac problems during follow-up; (7) assess the role of fibrosis on CMR (T1 mapping with evaluation of extracellular volume) after cardiotoxic radiation therapy or systemic therapy in predicting the incidence of cardiotoxicity; (8) detect the incidence of acute asymptomatic pericarditis after radiation therapy, measured on CMR; (9) investigate if the area of the oedema on CMR correlates with RT dose distribution; and (10) assess the incidence of myocardial oedema on CMR (T2 mapping) after radiation therapy and cardiotoxic systemic therapy, measured on CMR and ECHO over the time window of 12 months from the end of radiation therapy.

### Statistical Analysis

#### Sample Size Calculation

Sample size of this multicenter study is evaluated based on feasibility. We hypothesize to be able to enroll 150 patients satisfying the enrollment criteria and giving consent to participate into the study. We base calculations on the primary end point.

Table 1 summarizes the effect size for the primary end point that can be detected for the expected sample size, given a power of 80% and different rates of prevalence of myocardial oedema after cardiotoxic systemic therapy or radiation therapy.

We use an alpha level of 10% one-sided test, given the lack of strong evidence in the literature (“early evidence” study). We used Stata 15 (Stata Corp) for computation.

**Table 1:** Effect size computation assuming a rate of cardiac toxicity at 12 months of p1=17% in the cohort without oedema at magnetic resonance. Power 80%, alpha (2-sided) 20%.

Hypothesized points with MR^a^ oedema at the end of RT^b^, %	Detectable effect size in the oedema population, %
5	52 (25)
10	43 (26)
15	39 (22)
20	37 (20)
25	35 (18)

^a^MR: magnetic resonance.

^b^RT: radiotherapy.

#### Planned Analysis

Data at enrollment will be described as mean and standard deviation or median and 25th-75th percentiles if continuous and as counts and percent if categorical. They will be compared between groups of patients with and without oedema at the end of RT with the Student *t* test (or the Mann Whitney U test, based on the distribution) and the Fisher exact test, respectively.

#### Analysis of the Primary End Point

The number of patients with cardiac toxicity at 12 months will be compared between groups with the Fisher exact test. The mean difference in proportions of cardiac toxicity at 12 months and its 80% confidence interval will be reported. Logistic regression will be used to adjust for potential confounders. We do not expect losses to follow-up. A sensitivity analysis will classify them as no cardiac toxicity patients. If the mortality or losses to follow-up are above 10%, we will also compare groups using survival analysis methods to account for the different follow-ups between patients, using the same strategies.

#### Analysis of the Secondary Objectives

The data will be compared as described above. The time-to-event end points will be compared using survival analysis methods. The association of changes in biomarkers and changes in cardiac function will be assessed with linear regression models. Data transformation will be applied as needed. The details are provided in Table 2.

Multivariable analysis to identify potential predictors of cardiac toxicity will be performed, while considering a predictor-to-events ratio of 1:10 to avoid overfitting.

**Table 2:** Analysis of the secondary end points.

Secondary end point	Analysis
Detect GLS^a^ decrease of >15% from baseline, measured on ECHO^b^ over the time window of 12 months.	The number of patients with a >15% decrease will be compared between groups with the Fisher exact test. The mean difference in proportions at 12 months and its 80% CI will be reported.
See if the changes in biomarkers will correlate with LVEF^c^ measurements, assessed by ECHO and CMR^d^.	The association of changes in biomarkers and LVEF will be assessed with a linear regression model, while adjusting for oedema.
See if the changes in biomarkers will correlate with GLS measurements, assessed by ECHO.	The association of changes in biomarkers and GLS will be assessed with a linear regression model, while adjusting for oedema.
Compare the time to biomarkers’ positivity to the time to decrease in GLS >15% or decline in LVEF ≥10% in points with a final LVEF of <53% measured on ECHO.	The times will be compared with the Mann Whitney U test.
Find out if patients with increased baseline biomarkers will develop cardiotoxicity; identify predictors of cardiotoxicity by multivariable analysis.	A univariable and multivariable logistic model will be used.
Detect MACE^e^ (defined as acute myocardial infarction, hospitalization due to heart failure, atrial flutter or fibrillation, and ventricular tachycardia) or death due cardiac problems during follow-up.	The rate of each overall MACE and that of each event will be computed per 100 person-year with 80% CI. Kaplan Meier curves will be plotted.
Assess the role of fibrosis on CMR (T1^f^ mapping with evaluation of extracellular volume) after cardiotoxic radiation therapy or systemic therapy in predicting the incidence of cardiotoxicity.	A univariable and multivariable logistic model will be used.
Detect incidence of acute asymptomatic pericarditis after RT^g^, measured on CMR.	The proportion of patients with acute asymptomatic pericarditis and 80% CI will be computed.
Investigate if the area of the oedema on CMR correlates with RT dose distribution.	The Spearman correlation coefficient and 80% CI will be computed.
To assess the incidence of myocardial oedema on CMR (T2^h^ mapping) after radiation therapy and cardiotoxic systemic therapy measured on CMR and ECHO over the time window of 12 months from the end of radiation therapy.	The proportion of patients with oedema and 80% CI will be computed.

^a^GLS: global longitudinal strain.

^b^ECHO: echocardiography.

^c^LVEF: left ventricular ejection fraction.

^d^CMR: cardiac magnetic resonance.

^e^MACE: major cardiovascular events.

^f^T1: longitudinal relaxation time.

^g^RT: radiotherapy.

^h^T2: transverse relaxation time.

## Results

Recruitment started in September 2020. The results of this study will not be published until data are mature for the final analysis of the primary study end point.

## Discussion

### Summary

CardioTox Breast is a multicenter prospective longitudinal study testing the role of CMR in predicting cardiotoxicity in patients with breast cancer. The study is designed to combine both cardiac imaging information regarding potential early myocardial dysfunction and anatomical coronary changes, as well as variations in circulating cardiac damage biomarkers.

We will investigate the effects of systemic therapy and radiation therapy on myocardial function and structure, thus providing additional evidence on whether CMR is the optimal screening tool for cardiotoxicity. CMR is very promising for assessing the function and structure of the cardiovascular system and is starting to be investigated further in prospective studies [28].

### Conclusions

Cardiotoxicity can affect the quality of life of breast cancer survivors, whose numbers are increasing. It is important to distinguish high-risk patients who need intensive cardiovascular screening during and after cardiotoxic treatment.

Cardiotox Breast results should improve the prediction and prevention of potential lesions to normal cardiac tissue and ultimately enhance patients’ care and quality of life.

To our knowledge, this study is one of the first longitudinal studies with the primary aim of identifying any change in cardiac imaging (based on CMR and ECHO) and circulating biomarkers to predict the incidence of cardiotoxicity.

## Abbreviations

BNP: brain natriuretic peptide
CMR: cardiac magnetic resonance
CT: computed tomography
ECHO: echocardiography
ECOG: Eastern Cooperative Oncology Group
FISP: fast imaging with steady-state free precession
GLS: global longitudinal strain
hCG: human chorionic gonadotropin
HER2: human epidermal growth factor receptor 2
HF: heart failure
hs-CRP: high-sensitivity C-reactive protein
LVEF: left ventricular ejection fraction
RT: radiotherapy
